# Livestock adaptation to climate

**DOI:** 10.1093/af/vfad048

**Published:** 2023-10-13

**Authors:** Norman H Casey

**Affiliations:** Department of Animal Science, University of Pretoria, Pretoria, South Africa

## Change and Adaptation

Change and adaptation are the phenomena of life. Throughout the existence of life on earth, biological systems have adapted and changed physiologically and morphologically to acquire a homeostasis most suitable for the demands of the environment. The genetic composition is interchangeable among organisms, and the expressions of traits are the response genes in combination to biological requirements. Human interventions changed a plethora of gene expressions by direct selection including hunting, domestication, and selecting for desirable traits. Human interventions on the biological world are moving rapidly from direct to indirect interventions through overutilization of resources and secondary effects of industrial development on the earth’s ecosystems.

Extensive livestock farming has developed to having a footprint in virtually every climate zone with the subtropics and temperate zones being the most favorable. The subtropics between 30 and 34 degrees north and south of the equator are accommodating for large and small animal species. The subtropics however have a range of ecological zones and microclimates shaped by geological and atmospheric features. The range of ecological zones includes extensive rangeland varying from high rainfall grasslands to semidesert scrub regions; dense and extended savannas, summer rainfall, and Mediterranean winter rainfall; summer mean temperature of 27 °C reaching >40 °C, while the winter daytime temperature range is 5 to 12 °C. Winter snowfalls can occur at higher altitudes as far north as the Tropic of Capricorn in the Southern Hemisphere. Periodic, prolonged droughts occur throughout the subtropical zone, which is influenced by the El Niño–La Niño phenomenon that occurs in the southeastern Pacific Ocean.

This issue of *Animal Frontiers*, Livestock Adaptation to Climate, focuses on selected aspects of extensive livestock production: grassland and savanna, genetics of small stock and large stock, nutrition, and product quality.

## Subtropical Grasslands

The extensive grasslands and savanna of the summer rainfall regions have the greatest biomass yield. Under natural, unfenced grazing systems as with game and nomadic farmers where low input management is applied, spontaneous natural management practices evolve. The major physical threats are fire that destroys but allows for rejuvenation, over-usage, or drought.

The presentations offer retrospective analyses and prospective views on managing livestock production in terms of grasslands, animal genetics, nutrition, and meat quality. The subtropical grasslands and water are the critical resources of the subtropical zone.

Grassland management is addressed in terms of expected changing climatic conditions. In Resilience of warm-season (C_4_) grasses under challenging environmental and management conditions (Vendramini et al., 2023), it was noted that warm-season C_4_ grasses are the main source of nutrients for ruminant livestock, and that climate change may favor their expansion and displace C_3_ grasses. Three distinct physiological pathways and decarboxylation enzymes occur in C_4_ grass subtypes that are responsible for the adaptability of the subtypes to different edaphic-climatic regions. Although C_4_ grasses resilient under stressful defoliation management, anatomical and physiological differences can affect the resilience of these species to management practices. Nitrogen is usually the most limiting nutrient for growing C_4_ grasses, which combined with defoliation intensity and frequency may dictate the expansion and perennation of different C_4_ grass species in newly populated areas. In addition to superior forage production, C_4_ grasses have the potential to increase CO_2_ mitigation due to the efficient photosynthetic pathway and superior and superior biomass accumulation. A greater proportion of the biomass accumulation in C_4_ grasses occurs below ground, increasing C concentration in the soil and potential C sequestration. In summary, the expansion of C grasses in the world due to climate change has potential to increase forage production for livestock and mitigate greenhouse emissions in agriculture systems.

Kirkman et al. (2023) in Future-proofing extensive livestock production in subtropical grasslands and savannas presented the view that proactive future-proofing strategies could counteract the expected negative impacts of climate change on extensive livestock production in subtropical grasslands and savannas. These strategies would include breeding adapted livestock, in particular small animals to ensure animal health and performance. Grassland and savanna grazing management to ensure an adequate supply of the highest-quality wet-season forage and adequate volumes of dry-season forage is critically important for extensive livestock production under adverse conditions.

In addition to monitoring the changing profiles of C_4_ and C_3_ grass species, and being proactive in breeding adapted livestock and managing the grasslands and savannas, Maroto-Molina et al. (2023) in Matching livestock to landscape: a proposal of metrics to phenotype grazing distribution from GNSS collar data advocated the value of metrics, in particular remote sensing in developing management practices matching livestock species and grassland environments.

## Genetic Adaptations

The genetics of livestock suitable for the subtropical regions range from livestock that had adapted to the environment most likely by natural selection to decisive selection, crossbreeding, and a developing practice of gene insertion. Examples of adapted sheep are indigenous fat-tailed and predominantly hair breeds. However, changing economic objectives caused producers to select for or incorporate genes from other breeds expressing economic traits.

Natural selection for adaptive traits proceeds unabatedly, whereas planned selection and crossbreeding have evolved to technology-based. These aspects of applying livestock genetics for traits suitable for production and economic traits in the subtropics are addressed in the articles.

### Sheep

Cloete et al. (2023) noted in their article Breeds and lines of sheep suitable for production in challenging environments that unimproved, indigenous, fat-tailed sheep breeds and their composites outperformed commercial breeds for fitness traits, such as resistance to ticks and responses to heat stress. The unimproved indigenous breeds are historical breeds that had developed in southern Africa largely under nomadic farming systems of the Koi people who had inhabited the regions before Europeans had arrived in southern Africa.

Exotic livestock, for example, Merino sheep, were unadapted to the environment and were consequently susceptible to local parasites and environmental stressors. However, planned breeding and selection lead to breeds becoming tolerant to local stressors and hence indigenized. This is the positive of genotype by environment interaction (Nel et al., 2023). Additive gains resulting from genetic selection were reported for Merino lines resisting challenges from internal parasites, blowfly strike, and sheep lice. Lamb survival benefitted from selection for number of lambs weaned per ewe mated, being able to resist cold stress during a winter lambing season. Variation between breeds as well as between animals within breeds can thereby be exploited to allow the better adaptation of sheep to challenging environments. Nel et al. (2023) in Challenges and strategies for genetic selection of sheep better adapted to harsh environments noted that health and fitness traits are likely to remain difficult to measure, and new protocols should be combined with other developments in animal breeding such as genomic selection for worthwhile genetic gain.

### Goats

Angora and meat-producing goats play an important role in food security and sustainable livelihoods of livestock producers in South Africa. To maintain their role, it is important to select animals that can thrive in the harsh South African climate, especially under the envisioned climate change conditions. Although a number of phenotypes have been identified as selection criteria for adaptation, including improved fitness, growth, disease resistance, and heat tolerance, most of these have limitations such as being low heritable or difficult to measure. Visser and Snyman (2023) in Incorporating new technologies in breeding plans for South African goats in harsh environments advocate the application of new technologies in breeding and selection since technology is underutilized in goats in general, and especially in South African goats. The use of validated causative mutations could assist in the genetic improvement of these traits, and together with the systematic collection of relevant phenotypes can be used to improve the rate of animal improvement.

### Breeding to mitigate water stress

The intension of selective breeding for adaptive traits in livestock, as with grasses, is to enhance the expression of morphological and physiological traits favoring their tolerances of environmental stressors. As the availability of water can be a limiting factor in the harsher environments in the subtropics, Pérez et al. (2023) proposed in Mitigation and animal response to water stress in small ruminants that mitigation strategies to water stress will be needed to maintain animal production. Since water stress is usually related to heat stress and low intake separating these into distinct effects could be difficult. The authors maintained that small ruminants are able to better adapt to water stress than other species, especially breeds from regions under harsh conditions, and added that since breeding strategies could be difficult to implement, an option would be to apply omics technologies that can help to unravel the biological mechanisms involved in water stress adaptation.

### Cattle and buffalo

In Development of genetically improved tropically adapted cattle, Marchioretto et al. (2023) noted that improving local productivity in tropical regions promotes food security and livelihoods. To support this, a strategic exploration of traits of interest from distinct genetic groups would be a valuable tool to promote sustainability of livestock systems. It would require well-structured genetic improvement programs to avoid the opposite effect. In this respect, a broad approach should be considered and ideally accommodate the local necessities and conditions where they are being implemented.

In a similar vein, technology should be employed in selecting buffalo for heat tolerance. The HSP70 gene expression is an excellent biomarker for heat stress and thermotolerance in buffaloes. Furthermore, there are indications that thermotolerance could differ between buffalo breeds. By example, the Philippine Native Water Buffalo is more adaptive to heat stress, hence highly thermotolerant. Foreign breeds of buffaloes moved to the Philippines have developed adaptability to the tropical climate. In Development of adaptability of foreign breeds of water buffalo in Philippine Tropical Climate, Maylem (2023) advocated that thermotolerant and productive buffalo breeds should be a priority in breeding and genetic selection for improved animal production.

### Heat stress and nutrition

The nutrition of ruminants for their entire production cycle in the harsh environments relies on the available dry matter (DM) yield, DM nutrient value that can vary by locality and season, and the demands on their physiology in the productive phases (breeding, growing, yielding products). Heat stress is the most critical environmental stressor that can impact widely on production performance: fertility, growth, health, and product quality. Ameliorating heat stress through animal husbandry practices and adaptive genetics is presented in Impact of heat stress on ruminant livestock production and meat quality, and strategies for amelioration (Chauhan et al., 2023).

The impact of heat on metabolism was addressed in Physiological impact of amino acids during heat stress in ruminants (Loor et al., 2023). High seasonal heat and humidity decrease postruminal nutrient supply, deteriorate the ruminal and intestinal mucosa, and facilitate translocation of bioactive molecules into the bloodstream. Inflammation, oxidative stress, and misfolding of cellular proteins are physiological hallmarks of stress, including heat and humidity. These processes divert amino acid use away from productive purposes. Heat-stressed ruminants could benefit from increases in postruminal supply of arginine, cysteine, leucine, lysine, and methionine. One-carbon metabolism generates antioxidants from several indispensable and nondispensable amino acids as well as folic acid, choline, and betaine. “Omics” tools are allowing discovery of physiological mechanisms that can be manipulated via the supply of specific amino acids to alleviate the impact of heat stress.

## Conclusion

This issue of *Animal Frontiers*, Livestock Adaptation to Climate in association with the South African Society for Animal Science, presents a collective of practical and scientific insight into extensive livestock production in the subtropics.

*Conflict of interest statement.* None declared.

## About the Author

**Norman Casey**, DSc (Agric), is an emeritus professor in Animal Science at the University of Pretoria. He is a registered professional animal scientist with the South African Council for Natural Scientific Professions, Pr. Sci. Nat. (Anim. Sci.). He practices in livestock production physiology, though his academic work extends into associated fields, including tertiary education in the broader field of livestock production. He has held numerous offices in academia and in the scientific community including president of the statutory South African Council for Natural Scientific Professions and of the World Association for Animal Production.
